# Biologic Therapy in Ulcerative Colitis: A Retrospective Real-World Study in a Brazilian Tertiary University Hospital

**DOI:** 10.7759/cureus.108850

**Published:** 2026-05-14

**Authors:** Helena M Varandas, Ana Clara M Varandas, Cintia M Clemente

**Affiliations:** 1 School of Medicine, University of Brasília, Brasília, BRA; 2 School of Medicine, Catholic University of Brasília, Brasília, BRA; 3 Department of Gastroenterology, Hospital Universitário de Brasília, Brasília, BRA

**Keywords:** biologic therapy, epidemiology, infliximab, treatment outcome, ulcerative colitis, vedolizumab

## Abstract

Background: This study aimed to describe the demographic and clinical characteristics, biologic exposure history, switching patterns, treatment disruptions, selected complications/extraintestinal manifestations, disease extent, and chart-documented symptomatic status of patients with ulcerative colitis (UC) exposed to biologic therapy at a Brazilian tertiary university hospital.

Methods: This was a retrospective descriptive chart review using Aplicativo de Gestão para Hospitais Universitários/Empresa Brasileira de Serviços Hospitalares (AGHU/EBSERH) electronic medical records. Adults aged 18-80 years with confirmed UC and current or previous chart-documented biologic exposure for UC, followed between June 2022 and January 2024, were included. Data were abstracted and verified from August 2023 to July 2024. Variables included demographics, UC extent, biologics used, switching patterns and recorded reasons, treatment interruptions or discontinuation, selected complications and extraintestinal manifestations, and chart-documented symptomatic status. Because standardized disease activity indices, biomarkers, endoscopic assessments, and steroid-use data were not consistently available, symptomatic status was based on routine physician documentation. Analyses were descriptive; Wilson 95% confidence intervals (CIs) were calculated for key proportions.

Results: Among 132 UC outpatients followed during the study period, 127 met protocol-defined eligibility criteria after exclusions, and 34/127 (26.8%; 95% CI, 19.8%-35.1%) had current or previous biologic exposure. Most biologic-exposed patients were female (20/34, 58.8%) and lived in the Federal District (28/34, 82.4%). Infliximab was the most frequently documented biologic exposure (30/34, 88.2%; 95% CI, 73.4%-95.3%), followed by vedolizumab (13/34, 38.2%; 95% CI, 23.9%-55.0%) and adalimumab (8/34, 23.5%; 95% CI, 12.4%-40.0%). Thirteen patients (13/34, 38.2%; 95% CI, 23.9%-55.0%) had at least one biologic switch; allergic reactions (9/34, 26.5%; 95% CI, 14.6%-43.1%) and lack of response documented in the chart (4/34, 11.8%; 95% CI, 4.7%-26.6%) were the most common recorded reasons. Chart-documented symptomatic improvement or absence of gastrointestinal symptoms was recorded in 22/34 patients (64.7%; 95% CI, 47.9%-78.5%). Pancolitis was the most frequent disease extent (19/34, 55.9%; 95% CI, 39.5%-71.1%).

Conclusion: In this single-center Brazilian tertiary-care cohort, infliximab was the most frequent chart-documented biologic exposure, and biologic switching was common. Although many patients had chart-documented symptomatic improvement or absence of gastrointestinal symptoms, these findings should be interpreted cautiously because the study was retrospective and descriptive, and symptomatic status was not assessed using standardized disease activity indices or objective treat-to-target measures.

## Introduction

Ulcerative colitis (UC) is a chronic inflammatory bowel disease characterized by continuous mucosal inflammation that begins in the rectum and may extend proximally. In Brazil, population-level data suggest an increasing burden of inflammatory bowel diseases in the public healthcare system, reinforcing the need for robust long-term care strategies [1,2].

Current treatment goals extend beyond symptom control, emphasizing treat-to-target strategies that integrate patient-reported outcomes with objective inflammation control [3]. Parallel to this shift, the therapeutic armamentarium has expanded, including anti-tumor necrosis factor (anti-TNF) agents and gut-selective integrin blockade, which represent key guideline-supported biologic classes for moderate-to-severe disease, with positioning guided by contemporary clinical guidelines [4-6]. However, real-world effectiveness, tolerability, and therapy sequencing can vary across settings, particularly where care pathways and access constraints influence long-term management.

Against this background, this study aimed to describe the demographic and clinical characteristics, biologic exposure history, switching patterns and recorded reasons, treatment disruptions, selected complications and extraintestinal manifestations (EIMs), disease extent, and chart-documented symptomatic status of biologic-exposed UC patients followed in routine tertiary-care practice at a Brazilian university hospital. The objective was descriptive and was not intended to evaluate comparative effectiveness, formal remission, mucosal healing, or structured longitudinal treatment outcomes.

## Materials and methods

Study design and setting

This retrospective descriptive chart review was conducted in the tertiary outpatient inflammatory bowel disease clinic of Hospital Universitário de Brasília-Empresa Brasileira de Serviços Hospitalares (HUB-EBSERH), a Brazilian tertiary university hospital affiliated with the University of Brasília, Brasília, Federal District, Brazil. The study was designed to describe chart-documented biologic exposure and selected clinical variables in routine care, not to conduct repeated-measures, time-to-event, or comparative effectiveness analyses.

Data source and periods

Data were obtained exclusively from Aplicativo de Gestão para Hospitais Universitários (AGHU)/EBSERH electronic medical records. The eligible outpatient follow-up period was June 2022 to January 2024. Data abstraction and verification were performed from August 2023 to July 2024. Because the cohort included patients with current or previous biologic exposure, biologic use was handled descriptively as chart-documented “ever exposure” during the available record, rather than as an inception cohort beginning at biologic initiation.

Participants

Adults aged 18-80 years with a confirmed UC diagnosis and documentation of current or previous biologic exposure for UC were eligible. UC diagnosis and disease extent were abstracted from gastroenterology specialist records and available endoscopic/histopathologic documentation when recorded in the medical chart. According to the institutional protocol approved for data collection, patients were excluded if they (i) did not have clinical records, (ii) were pregnant at screening, (iii) had active tobacco or alcohol use documented at screening, or (iv) lacked documented biologic exposure. These exclusions were protocol-defined and were not post hoc analytic decisions. However, because smoking and alcohol use may be clinically relevant in UC populations, these exclusions may reduce external validity and were considered in the interpretation of the findings.

Variables and outcomes

We extracted sex, age group, marital status, residence, UC extent as recorded in the chart, biologics used (ever), switching events and recorded reasons, treatment interruptions or discontinuation, selected complications and EIMs, and chart-documented symptomatic status. Biologic switching was defined as any chart-documented replacement of one biologic agent by another during the available record. Recorded reasons for switching or disruption were abstracted as documented by the treating team and were not assumed to be mutually exclusive unless clearly stated in the chart. Missing information was reported as “not recorded” or “not specified,” and no imputation was performed.

Because standardized disease activity indices were not consistently available, and biomarkers, endoscopic assessment, and steroid-use data were incompletely documented, we did not use a validated clinical response endpoint. Instead, chart-documented symptomatic status was pragmatically defined as physician-documented symptomatic improvement or absence of gastrointestinal symptoms during routine follow-up. This variable should not be interpreted as a formal clinical response, remission, mucosal healing, or treat-to-target disease control.

Adverse events, complications, and EIMs were recorded as documented during follow-up. Causality was not inferred unless explicitly stated in the medical record. Conditions such as anemia, toxic megacolon, and low-grade tubular adenoma were treated as relevant complications/events rather than automatically classified as EIMs.

Statistical analysis

All statistical analyses were performed using SPSS version 26.0 (IBM Corp., Armonk, NY, USA) and Microsoft Excel (Microsoft Corp., Redmond, WA, USA). Given the descriptive design, small biologic-exposed subgroup, incomplete standardized outcome ascertainment, and lack of prespecified comparative hypotheses, no inferential hypothesis testing was performed. Categorical variables are presented as absolute frequencies and percentages using n/N (%) where appropriate. Wilson 95% confidence intervals (CIs) were calculated for key descriptive proportions to improve interpretability. No missing-data imputation was performed.

Ethics

The study was approved by the institutional Research Ethics Committee (CAAE: 79471923.2.0000.5558; approval number: 6.945.187) and conducted in accordance with local regulations.

## Results

Population

Among 132 UC outpatients followed during the study period, 127 met eligibility criteria after protocol-defined exclusions (pregnancy, n = 3; active tobacco or alcohol use, n = 2). Of these, 34/127 (26.8%; 95% CI, 19.8%-35.1%) had current or previous chart-documented biologic exposure and composed the biologic-exposed subgroup analyzed descriptively.

Demographic profile and disease extent

Most patients were female (20/34, 58.8%). Age distribution was 20-40 years (14/34, 41.2%), 40-60 years (14/34, 41.2%), and 60-80 years (6/34, 17.6%); no patient was younger than 20 years. Regarding marital status, 44.1% (15/34) were single, 20.6% (7/34) were married, 2.9% (1/34) were in a stable union, 2.9% (1/34) were widowed, and 29.4% (10/34) had no marital status recorded.

Residence was predominantly the Federal District (28/34, 82.4%), with the remainder residing in other regions. Pancolitis was the most frequent extent (19/34, 55.9%), followed by left-sided colitis (7/34, 20.6%). Proctosigmoiditis (3/34, 8.8%), rectosigmoiditis (1/34, 2.9%), and extensive colitis (1/34, 2.9%) were less common; extent was not specified in 3/34 (8.8%). The demographic characteristics and UC extent of the biologic-exposed patients are summarized in Table 1.

**Table 1:** Demographic characteristics and ulcerative colitis extent of biologic-exposed patients Demographic characteristics and ulcerative colitis extent of biologic-exposed patients (N = 34). Percentages are based on N = 34; percentages may not sum to 100% due to rounding.

Characteristic	n	%
Sex		
Male	14	41.2
Female	20	58.8
Age group (years)		
20-40	14	41.2
40-60	14	41.2
60-80	6	17.6
Residence		
Federal District (DF)	28	82.4
Other Brazilian states	6	17.6
Marital status		
Single	15	44.1
Married	7	20.6
Stable union	1	2.9
Widowed	1	2.9
Not reported	10	29.4
Ulcerative colitis extent (as recorded)		
Proctosigmoiditis	3	8.8
Rectosigmoiditis	1	2.9
Left-sided colitis	7	20.6
Extensive colitis	1	2.9
Pancolitis	19	55.9
Not specified	3	8.8

Biologic exposure, switching, and treatment disruptions

Infliximab was the most frequently documented biologic exposure (30/34, 88.2%; 95% CI, 73.4%-95.3%), followed by vedolizumab (13/34, 38.2%; 95% CI, 23.9%-55.0%) and adalimumab (8/34, 23.5%; 95% CI, 12.4%-40.0%). Thirteen patients (13/34, 38.2%; 95% CI, 23.9%-55.0%) had at least one biologic switch. The most commonly recorded reasons were allergic reactions (9/34, 26.5%; 95% CI, 14.6%-43.1%) and lack of response documented in the chart (4/34, 11.8%; 95% CI, 4.7%-26.6%). Across the cohort, the most frequent switching sequence was infliximab to vedolizumab (7/34, 20.6%).

Treatment interruptions due to delayed medication availability were documented in 2/34 (5.9%). Medication loss was recorded in 1/34 (2.9%). One patient (1/34, 2.9%) discontinued biologic therapy on their own. The biologic therapies used, switching patterns, and treatment disruption events are summarized in Table 2.

**Table 2:** Biologic therapies used and switching or disruption patterns among ulcerative colitis patients Biologic therapies used and switching or disruption patterns among ulcerative colitis patients (N = 34). Percentages are based on N = 34. Biologic exposure and recorded reasons or events are not mutually exclusive; percentages may not sum to 100%.

Characteristic	n	%
Biologics used (ever)		
Infliximab	30	88.2
Vedolizumab	13	38.2
Adalimumab	8	23.5
Biologic switching patterns		
Patients with one or more biologic switch	13	38.2
Infliximab to vedolizumab	7	20.6
Adalimumab to vedolizumab	1	2.9
Infliximab to adalimumab	2	5.9
Adalimumab to infliximab	1	2.9
Infliximab and/or adalimumab to vedolizumab	2	5.9
Recorded reasons/events (as documented)		
Allergic reactions	9	26.5
Lack of response to treatment	4	11.8
Medication loss by the patient	1	2.9
Self-discontinuation (patient decision)	1	2.9
Treatment interruption due to delay in medication availability	2	5.9
Active tuberculosis diagnosed during infliximab therapy	1	2.9

Chart-documented symptomatic status

Chart-documented symptomatic improvement or absence of gastrointestinal symptoms during biologic therapy was recorded in 22/34 patients (64.7%; 95% CI, 47.9%-78.5%). This variable was based on routine physician documentation and was not equivalent to a validated clinical response or remission endpoint.

Complications, EIMs, and relevant events

EIMs and relevant complications/events included primary sclerosing cholangitis (5/34, 14.7%), anemia (3/34, 8.8%), pyoderma gangrenosum (2/34, 5.9%), spondyloarthritis (2/34, 5.9%), toxic megacolon (1/34, 2.9%), and low-grade tubular adenoma (2/34, 5.9%). Hand osteoarthritis was documented in one patient (1/34, 2.9%) but was not classified as a typical UC-related EIM. One case of active tuberculosis diagnosed during infliximab therapy was documented; prior latent tuberculosis status could not be confirmed from the available data (Table 3).

**Table 3:** Complications, extraintestinal manifestations (EIMs), and clinical response in biologic-exposed ulcerative colitis patients Complications, EIMs, and chart-documented symptomatic status in biologic-exposed ulcerative colitis patients (N = 34). Chart-documented symptomatic status was pragmatically defined as physician-documented symptomatic improvement or absence of gastrointestinal symptoms during routine follow-up, because validated disease activity indices were not consistently available in the medical records. Percentages are based on N = 34. Complications and EIMs are not mutually exclusive; percentages may not sum to 100%.

Characteristic	n	%
Complications, extraintestinal manifestations (EIMs), or relevant events		
Primary sclerosing cholangitis (PSC)	5	14.7
Pyoderma gangrenosum	2	5.9
Spondyloarthritis	2	5.9
Hand osteoarthritis	1	2.9
Anemia	3	8.8
Toxic megacolon	1	2.9
Low-grade tubular adenoma	2	5.9
Chart-documented symptomatic status during biologic therapy		
Symptomatic improvement or absence of gastrointestinal symptoms	22	64.7
Persistent symptoms or no documented symptomatic improvement	12	35.3

## Discussion

In this retrospective descriptive chart review from a Brazilian tertiary university hospital, infliximab was the most frequently used biologic, biologic switching was common, and chart-documented symptomatic improvement or absence of gastrointestinal symptoms was recorded in most biologic-exposed patients. Within the scope of the present study, these findings should be interpreted as a descriptive overview of biologic-treated UC in routine care rather than as evidence of comparative effectiveness between agents, formal clinical response, remission, mucosal healing, or treat-to-target disease control.

The predominance of infliximab in this cohort is compatible with the established role of advanced therapies in the management of moderate-to-severe UC and with contemporary guideline recommendations that include biologic therapy among standard treatment options in appropriate clinical scenarios [4-6]. At the same time, because the present study was retrospective, descriptive, and based on a small biologic-exposed subgroup, the high frequency of infliximab use in our sample should be understood primarily as a local treatment pattern rather than as evidence of superiority over other biologic agents.

Biologic switching was observed in 13 of 34 patients, and the most commonly documented reasons were allergic reactions and lack of response. This finding is clinically relevant because it highlights that biologic therapy in UC is often dynamic in routine practice and may require modification over time according to tolerability and clinical evolution. This interpretation is consistent with observational evidence showing that biologic persistence and sequencing vary in real-world inflammatory bowel disease care [7,8]. However, our data do not support direct comparisons between specific agents, since the study was not designed for comparative effectiveness analysis and no inferential statistical testing was performed [7,8].

The rate of chart-documented symptomatic improvement should also be interpreted with caution. In this study, symptomatic status was pragmatically defined on the basis of routine medical-record documentation because validated disease activity indices were not consistently available. Accordingly, the observed improvement is clinically informative as a real-world chart-documented finding, but it should not be interpreted as equivalent to validated clinical response, formal remission, mucosal healing, or complete target-based disease control. This distinction is particularly important in light of current treat-to-target concepts, which emphasize the integration of symptoms with objective measures of inflammation and disease activity [3]. In addition, this variable may be affected by subjective documentation bias, heterogeneous timing of follow-up, absence of a standardized baseline severity anchor, and incomplete availability of biomarkers, endoscopic findings, steroid-use data, or validated patient-reported outcomes.

Another relevant finding was the occurrence of one case of active tuberculosis diagnosed during infliximab therapy. Although only one event was documented, it warrants attention because anti-TNF therapy is a recognized risk context for tuberculosis reactivation and active tuberculosis, particularly in patients with inflammatory bowel disease receiving immunosuppressive treatment [9-11]. Because baseline latent tuberculosis status and concomitant immunosuppression details were not consistently available in the abstracted dataset, this event should be interpreted as a documented event during follow-up rather than as proof of causal attribution. In this regard, the event observed in our cohort reinforces the importance of rigorous screening and follow-up strategies in biologic-treated patients, especially when anti-TNF agents are used [9-11].

The burden of disease-related manifestations recorded in this cohort also deserves attention. Primary sclerosing cholangitis, pyoderma gangrenosum, spondyloarthritis, and anemia were documented in the biologic-exposed subgroup, reinforcing that UC may be associated with clinically relevant manifestations beyond luminal symptoms alone and may require multidisciplinary care according to the profile of each patient [12,13]. In addition, intestinal and colonic complications observed in this cohort, including toxic megacolon and low-grade tubular adenoma, further illustrate the complexity of follow-up in tertiary-care practice. However, because this study was descriptive and based on chart abstraction, it cannot determine whether these events were associated with biologic exposure, disease severity, referral patterns, or other clinical factors.

Treatment interruptions related to delayed medication availability, medication loss, and self-discontinuation were infrequent but clinically relevant. Even in a descriptive study, these events help contextualize biologic use in routine care because continuity and adherence are important components of long-term inflammatory bowel disease management, and inadequate adherence has been associated with worse clinical outcomes, including increased disease activity and higher relapse risk [14]. Taken together, these findings support the value of structured monitoring, careful documentation of treatment response and adverse events, and coordinated follow-up in biologic-exposed patients with UC in real-world practice [14].

The biologic distribution observed in this cohort is also coherent with the broader therapeutic evidence base in UC. Randomized trials demonstrated the efficacy of infliximab, vedolizumab, and adalimumab for moderate-to-severe UC [15-17], and a head-to-head randomized trial later showed vedolizumab to be superior to adalimumab with respect to clinical remission and endoscopic improvement, although not corticosteroid-free clinical remission [18]. These studies do not allow comparative conclusions within our sample, but they provide an appropriate evidence-based framework for understanding why multiple biologic agents appear in routine care and why switching between therapies is part of contemporary UC management [15-18].

Limitations

This retrospective single-center descriptive chart review included a small biologic-exposed subgroup, which limits the interpretation of less frequent events and reduces external validity. In addition, the study included patients with current or previous biologic exposure rather than a clearly defined inception cohort, which may introduce prevalent-user bias and limit temporal interpretation of biologic exposure, switching, and outcomes.

Chart-documented symptomatic status was based on routine medical-record documentation, since validated disease activity indices were not consistently available in the medical records. Biomarkers, endoscopic assessment, steroid-use data, baseline disease severity, disease duration, indication for biologic initiation, biologic dose optimization, therapeutic drug monitoring, concomitant immunomodulator or corticosteroid use, hospitalization history, and prior surgery were also not consistently documented. Therefore, the findings should be interpreted as descriptive real-world data from routine care rather than as formal clinical response, remission, mucosal healing, or treat-to-target outcomes [3].

Additional limitations include heterogeneous follow-up, absence of standardized timing for symptomatic assessment, possible documentation bias, variable misclassification, and confounding by indication. The protocol-defined exclusion of pregnant patients and patients with active tobacco or alcohol use may also have introduced selection bias and limited the representativeness of the cohort in relation to the broader population of biologic-treated patients with UC. Finally, because this study was descriptive and did not include inferential or time-to-event analyses, it cannot establish causality, compare the effectiveness of biologic agents, or determine whether adverse events and complications were attributable to biologic exposure.

## Conclusions

In this single-center Brazilian tertiary-care cohort of biologic-exposed UC patients, infliximab was the most frequently documented biologic exposure, and switching between biologics was common, mainly due to chart-documented allergic reactions and lack of response. Although chart-documented symptomatic improvement or absence of gastrointestinal symptoms was recorded in many patients, these findings should be interpreted cautiously because the study was retrospective and descriptive and did not use standardized disease activity indices or objective treat-to-target outcomes. The observed treatment disruptions, selected complications/EIMs, and one case of active tuberculosis diagnosed during infliximab therapy highlight the need for structured documentation, monitoring, and coordinated follow-up in real-world tertiary-care practice.
